# An exploration of sharps injuries within healthcare students at a UK university

**DOI:** 10.1177/17571774241238661

**Published:** 2024-03-14

**Authors:** K Hambridge, W Burt, G Bettache, M Keshishian, T Walvin, E Cozma

**Affiliations:** 1 School of Nursing and Midwifery, 6633 University of Plymouth, Plymouth, UK; 2 Bosvena Health, Plymouth, UK; 3 6633 University of Plymouth, Plymouth, UK

**Keywords:** Sharps, infection control, student, healthcare, nursing, dental, medical, podiatry, paramedicine, occupational therapy

## Abstract

**Background:**

There is evidence available worldwide that nursing, medical and dental students sustain sharps injuries during their programmes of study. However, there is lack of evidence and research relating to the many students of other healthcare professions who may encounter sharps instruments.

**Aim/objective:**

The aim of the study was to identify the extent, type and impact of sharps injuries sustained by pre-registration healthcare students.

**Methods:**

An online survey was administered to 3372 healthcare students at a University in the United Kingdom.

**Findings/results:**

Some healthcare students other than nursing, medical and dentistry had sustained a sharps injury. The most common device involved were glass ampoules. The common causes were equipment and carelessness. Some healthcare students sustained psychological impacts following the sharps injury.

**Discussion:**

Sharps injuries are common amongst some healthcare students and there is scope for more education for these groups of students relating to the risks, safe handling, reporting and prevention of sharps injuries.

## Background/literature review

The purpose of this study was to identify the extent, type and impact of sharps injuries sustained by pre-registration healthcare students within a university in the UK. Sharps injuries have been defined as needles, blades and other medical instruments used in healthcare which have the potential to cause a cutting or pricking injury (HSE, 2021).

Sharps injuries are a well-known risk in the health and social care sector. These types of injuries can have many consequences. Sharps contaminated with an infected patient’s blood can transmit more than 20 diseases, including hepatitis B, C and human immunodeficiency virus (HIV) (Cooke and Stephens, 2017). Because of this transmission risk, sharps injuries can cause worry and stress to the many thousands who receive them (HSE, 2021). These types of injuries can also have a huge psychological impact on the recipient and their families (RCN, 2009). This can include anxiety (Gershon et al., 2000), depression (Sohn et al., 2006) and post-traumatic stress disorder (Naghavi et al., 2013).

Amongst the numerous laws that relate to sharps usage and safety, the Health and Safety (Sharp Instruments in Healthcare) Regulations (2013) makes it clear that employers should make sure that the risks of sharps injuries are sufficiently regulated. This is significant as healthcare students spend a proportion of their learning potentially handling sharps. The key points to ponder for students are that there should be an avoidance of the superfluous use of sharps; that safer sharps should be accessible for usage; recapping should be prohibited; and medical sharps disposal containers should be utilised safely.

Studies specifically investigating sharps injuries within multiple healthcare students are sparse. Bhattarai et al. (2014) studied sharps injuries within healthcare students in Nepal and found that 42.8% (*n* = 90) had experienced at least one injury during the programme. Nursing students accounted for 70% (*n* = 63); 20% (*n* = 18) were medical students; and 10% (*n* = 9) were dental students. McCarthy and Britton (2000) identified similar student types when investigating sharps injuries within healthcare students in Canada and found that 82% involved dental students, 57% involved medical students and 27% involved nursing students.

Nursing students have been investigated for rates of sharps injuries in a recent systematic review and meta-analysis and evidence suggests that between 6–51% of populations worldwide have sustained a sharps injury (Chen and Zhang, 2021). Midwifery students are commonly amalgamated with nursing students within studies, and the range of sharps injuries is reported to be between 35.5% (Karadağ, 2010; Kursun and Arslan, 2014). When including medical students, the range is between 11% (Varsou et al., 2009) and 95% (Liyanage et al., 2012) and within dentistry students between 13% (Smith et al., 2006) and 43.1% (Fernandes et al., 2017). Information and evidence though are very limited regarding the extent, circumstances and reporting of sharps injuries in other undergraduate healthcare students (Hambridge et al., 2016; Marnejon et al., 2016) and this is the reason for investigating this population.

## Aims and objectives

The aim of the study was to identify the extent, type and impact of sharps injuries sustained by pre-registration healthcare students. The objectives were:

• To identify which healthcare students sustain sharps injuries
• To understand the extent of sharps injuries sustained by healthcare students
• To discover the type of sharps injury that healthcare students sustain
• To identify the impact of the sharps injury on the individual student

## Methodology and methods

### Study design

A cross-sectional survey was conducted.

### Participants and setting

Convenience sampling was utilised to recruit participants from one University in the UK. The inclusion criteria were students enrolled on pre-registration healthcare programmes including nursing, midwifery, medicine, dentistry, paramedicine, dietetics, physiotherapy, occupational therapy, optometry, podiatry and radiography. The programmes were studied at BSc and MSc level and lasted for 3–6 years depending upon the speciality and the programme.

### Materials and procedures

The questionnaire was based upon a previously validated and reliable tool (Hambridge et al., 2021) designed to explore sharps injuries within a nursing student population. The questionnaire was modified to incorporate the potential varied experiences within other healthcare students. The modified questionnaire was distributed to lecturers representing each student group to explore the face and content validity. Only minor changes were required. A small pilot study (*n* = 23) was completed with minor grammatical corrections suggested by healthcare students.

The questionnaire collected demographic and background data and then comprised of 17 questions (Box 1). Four questions were employed to investigate the impact of a sharps injury, including Post Traumatic Stress Disorder (PTSD). The Primary Care PTSD Screen (PC-PTSD Screen) (US Department of Veteran Affairs, 2013) was used and very slightly modified for this purpose of identifying potential PTSD following a sharps injury. Existing research proposes that the results of the PC-PTSD should seen as “positive” if a person answers “Yes” to any three items or more.

Box 1

1. Have you had a sharps injury in this current academic year?
2. How many sharps injuries have you had in this current academic year?
3. Please state the device(s) involved when you had the sharps injury (injuries).
4. Please indicate what procedure was happening when the sharps injury (injuries) occurred.
5. Please state what time of day or night the sharps injury (injuries) happened.
6. Please state what you consider were up to three potential ‘causes’ or ‘contributing factors’ of the sharps injury (injuries)
7. Were you being directly observed by your mentor, a Registered Nurse or a health professional, or University Lecturer at the time of the sharps injury (injuries)?
8. Please state if the sharp involved in the injury (injuries) was ‘used’ (contaminated) or ‘unused’ (sterile)
9. Please state the exact location where the sharps injury (injuries) occurred
10. Please state the ‘speciality’ of the placement where you had the sharps injury (injuries)
11. Please state if you reported the sharps injury (injuries)
12. If you did not report the sharps injury (injuries), please state the main reason why you did not report the sharps injury (injuries)
13. Please state which part of your body was injured when the sharps injury (injuries) occurred
14. In the month following the sharps injury (injuries) did you have nightmares about it or thought about it when you did not want to?
15. In the month following the sharps injury (injuries) did you try hard not to think about it or went out of your way to avoid situations that reminded you of it?
16. In the month following the sharps injury (injuries) were you constantly on guard, watchful or easily startled?
17. In the month following the sharps injury (injuries) did you feel numb or detached from others, activities or your surroundings?

The online questionnaire was created utilising the Jisc online survey tool (Joint Information Systems Committee) (Jisc, 2021). It was distributed via email to students enrolled on healthcare programmes in the last stages of their academic year, on or near completion of a clinical placement. The timespan for the distribution and collection of the questionnaires was between May and September 2022. Reminder emails to boost completion were sent four times.

### Ethics

The participants were informed that participation was entirely voluntary and that they had the right to withdraw from the study before the survey was submitted. Students were informed that participation in the study, or refusal to take part, would have no bearing at all on their progress within the programme which they were studying. There was no coercion or duress placed upon the participants in the light of the lecturer–student relationship which remained professional throughout.

The information given to potential respondents stated that due to the delicate nature of the study, counselling or support from their University services or GP should be sought if they felt that they had been psychologically harmed by the sharps injury. Because of the anonymous nature of surveys, it was not possible to ensure this had happened.

The information stated that responses were confidential and anonymous, and that the survey was not a test of knowledge but the researcher was interested in the honest views and opinions of the participant. It was stated that this study had been approved by the Ethics Committee at the University. Finally, potential respondents were informed that if they had any questions or concerns about the project, contact details of the researcher were provided.

### Analysis

Descriptive statistics were reported using frequency and percentages for categorical variables, and median, range and interquartile range for continuous variables. Either a Chi-squared test of independence or a Fisher’s exact test was applied for assessing associations between variables of interest, according as the assumptions underlying the respective test was satisfied.

## Results

### Response rate

The survey questionnaire was distributed to 3372 healthcare students and 724 responded giving an overall response rate of 21.47%. There was variation with the response rate between programmes (Table 1).

**Table 1.** Response rates of sharps injuries by study programme, in descending order.

Programme	Population	Number of responses	Response rate(%)
Midwifery	222	75	33.78
Nursing: mental health	165	55	33.33
Nursing: adult health	586	181	30.89
Nursing: child health	159	46	28.93
Paramedicine	207	49	23.67
Podiatry	69	16	23.19
Dentistry	342	78	22.81
Radiography	91	18	19.78
Medicine	740	141	19.05
Occupational therapy	236	24	10.17
Dietetics	123	12	9.76
Physiotherapy	210	17	8.1
Optometry	222	12	5.41
**Total**	**3372**	**724**	**21.47**

### Demographics

Most respondents were female (81%), aged between 20 and 29 years old (66%) and in the first 3 years of the programme (88%) (Table 2).

**Table 2.** Breakdown of respondents by gender, age and year of study.

	Total
Gender	
Female	585 (81%)
Male	134 (19%)
Other	2 (0.3%)
Prefer not to say	3 (0.4%)
**Total**	724 (100%)
Age	
17–19	81 (11%)
20–29	474 (66%)
30–39	114 (16%)
40–49	45 (6.2%)
50–59	6 (0.8%)
**Total** ^a^	720 (100%)
Year of study	
1st year	212 (29%)
2nd year	237 (33%)
3rd year	191 (26%)
4th year	48 (6.6%)
5th year	30 (4.1%)
6th year	6 (0.8%)
**Total**	724 (100%)

^a^4 respondents did not give data about their age.

### Incidence of sharps injuries

The incidence rate of sharps injuries within the last academic year amongst healthcare students was 13% (*n* = 93). Most injured students were aged between 20 and 29 (70% *n* = 65) and female (88% *n* = 82). Sharps injuries occurred more commonly in the first three academic years (28–29%) than in the sixth year (2.2%). When considering different healthcare programmes, podiatry had the highest prevalence (31% *n* = 5), followed by midwifery (21% *n* = 16). Students from four programmes did not sustain sharps injuries (Table 3).

**Table 3.** Prevalence rate of sharps injuries per programme.

Programme	Prevalence rate
Podiatry	31% (*n* = 5)
Midwifery	21% (*n* = 16)
Child nursing	17% (*n* = 8)
Paramedicine	16% (*n* = 8)
Adult nursing	14% (*n* = 25)
Medicine	12% (*n* = 17)
Mental health nursing	11% (*n* = 6)
Dentistry	9% (*n* = 7)
Occupational therapy	4.2% (*n* = 1)
Dietetics	0%
Optometry	0%
Physiotherapy	0%
Radiography	0%

### Number of sharps injuries

Most students (82.8% *n* = 77) who sustained an injury had one sharps injury per academic year, with 12.9% (*n* = 12) having two injuries.

### Devices that caused the injuries

Analysis of individual injuries showed 19 different devices which were involved in the production of a sharps injury. Glass ampoules (39.6% *n* = 40) were the most common, followed by hollow bore subcutaneous or intramuscular needles (17.8% *n* = 18). Interestingly one adult nursing student sustained an injury with a patient’s egg piercer (Table 4).

**Table 4.** Devices that produced the sharps injuries among healthcare students.

Devices	*n* (%)
Glass ampoule	40 (39.6%)
Needle (hollow bore) for subcutaneous/intramuscular injection	18 (17.8%)
Intravenous needle	14 (13.9%)
Scalpel/stitch cutter	7 (6.9%)
Suture needle	5 (5.0%)
Scissors	3 (3.0%)
Drawing up needle	2 (2.0%)
Blood collection needle	1 (1.0%)
Blood glucose lancet	1 (1.0%)
Clamp	1 (1.0%)
Dental burs	1 (1.0%)
Filter needle	1 (1.0%)
Insulin needles	1 (1.0%)
Local anaesthetic needle	1 (1.0%)
Nail nipper (podiatry)	1 (1.0%)
Patient’s egg piercer	1 (1.0%)
Razor	1 (1.0%)
Scaler tips	1 (1.0%)
Tablet cutter	1 (1.0%)
**Total**	**101 (100.0%)**

### Timing of sharps injury

Most sharps injuries (55.9% *n* = 52) occurred in the morning between 0600 and 1159, followed by 1200 and 1759 (31.2% *n* = 29) and 1800 and 2359 (11.8% *n* = 11).

### Main causes of the sharps injury

A total of 87 responses reported the main cause of the sharps injury were ‘the equipment’, ‘carelessness’ and ‘inexperience.’ A total of 10 potential causes were reported (Table 5).

**Table 5.** Prevalence of self-reported causes underlying sharps injuries, reported in terms of respondents.

Cause	Respondents (%)
The equipment you were using	20 (23.0%)
Your carelessness	20 (23.0%)
Your inexperience	14 (16.1%)
Your haste	13 (14.9%)
Lack of protection devices	7 (8.0%)
Lack of supervision	4 (4.6%)
Your lack of familiarity with the device	4 (4.6%)
Your heavy workload	3 (3.4%)
Your lack of sleep or tiredness	1 (1.1%)
Your stress levels	1 (1.1%)
**Total**	**87 (100.0%)**

### Prevalence of respondents observed during the injury

In total, more than one-third of students (36.6% *n* = 34) were not being observed when the sharps injury occurred. This included respondents from all the programmes where a healthcare student sustained a sharps injury, with the highest prevalence being occupational therapy (100% *n* = 1), podiatry (80% *n* = 4) and dentistry (71.43% *n* = 5) students.

### Used or clean sharp

Over a quarter of the sharps injuries (25.8% *n* = 24) were sustained with a sharp which had been used.

### Location of sharps injury

The most common location for a sharps injury to occur was in the ‘treatment room’ (21% *n* = 21), followed by the ‘patient’s bedside’ (16% *n* = 16) and a ‘clinic’ (15% *n* = 15). In total, there were 14 different locations where sharps injuries were reported (Table 6).

**Table 6.** Location of sharps injury.

Location of sharps injury	*n* (%)
Treatment room	21 (21%)
Patient’s bedside	16 (16%)
Clinic	15 (15%)
Theatre (including anaesthetic room, operating theatre and recovery)	11 (11%)
Education simulation environment	10 (10%)
Delivery room	7 (7%)
Patient’s own home	7 (7%)
Ambulance	4 (4%)
Drug storage facility	4 (4%)
Changing room of academy in hospital	1 (1%)
Chemotherapy unit	1 (1%)
Emergency department	1 (1%)
GP consultation room	1 (1%)
Public place where patient collapsed: restaurant	1 (1%)
**Total**	**100 (100%)**

### Specialty where injury occurred

The most common specialities where sharps injuries occurred were ‘medical placements’ (15.31% *n* = 15), ‘obstetrics/gynaecology/maternity’ (13.27%) and the education simulation environment (10.2% *n* = 10). In total, sharps injuries were reported in 20 specialities (Table 7).

**Table 7.** Distribution of specialities of students who suffered sharps injuries.

‘Specialty’ of the placement where you had the sharps injury (injuries)	*n* (%)
Medical ward/placement	15 (15.31%)
Obstetrics/gynaecology/maternity	13 (13.27%)
Education simulation environment	10 (10.2%)
Theatre (including anaesthetic room, operating theatre and recovery)	9 (9.18%)
Surgical ward/placement	8 (8.16%)
Emergency department	8 (8.16%)
Mental health	7 (7.14%)
Community hospital	6 (6.12%)
General practice	4 (4.08%)
Paediatrics	4 (4.08%)
Private clinic	3 (3.06%)
Oncology	2 (2.04%)
Outpatients department	2 (2.04%)
Ambulance, pre-hospital	1 (1.02%)
Dental clinic	1 (1.02%)
District nursing	1 (1.02%)
Hospice care	1 (1.02%)
Midwifery unit	1 (1.02%)
Out of hospital	1 (1.02%)
Paramedic placement	1 (1.02%)
**Total**	**98 (100%)**

### Part of body affected

The vast majority of injuries occurred to the hand (97.8% *n* = 91) although within dentistry one injury occurred to the lip and one to the arm.

### Reporting of the sharps injury

More than half of sharps injuries (53% *n* = 49) were not reported. Paramedicine (75% *n* = 6) and Dentistry (71% *n* = 5) had the highest report rates with Occupational Therapy (0% *n* = 1) having the lowest reporting rate (Table 8).

**Table 8.** Was the sharp injury reported.

Programme	Was the sharps injury reported?	
	No	Yes
Occupational therapy (*n* = 1)	1 (100%)	0 (0%)
Medicine (*n* = 17)	12 (71%)	5 (29%)
Podiatry (*n* = 5)	3 (60%)	2 (40%)
Midwifery (*n* = 16)	9 (56%)	7 (44%)
Nursing: adult health (*n* = 25)	14 (56%)	11 (44%)
Nursing: child health (*n* = 8)	4 (50%)	4 (50%)
Nursing: mental health (*n* = 6)	2 (33%)	4 (67%)
Dentistry (*n* = 7)	2 (29%)	5 (71%)
Paramedicine (*n* = 8)	2 (25%)	6 (75%)
**Total (*n* = 93)**	49 (53%)	44 (47%)

### The reason for not reporting of sharps injuries

The most common reason for not reporting the sharps injury included it being a ‘minor injury’ (36% *n* = 40) and being ‘unused or clean equipment’ (30% *n* = 34). In total, there were 13 reasons given for non-reporting (Table 9).

**Table 9.** Reasons for failing to self-report sharps injuries.

If not reported, why	*n* (%)
It was a minor injury	40 (36%)
It was ‘unused’ or clean equipment	34 (30%)
I was too embarrassed to report the injury (injuries)	9 (8%)
The patient was not infected	7 (6%)
I did not know how to report the injury (injuries)	5 (4%)
I was afraid to report the injury (injuries)	5 (4%)
I was worried reporting the injury would affect your assessment of competence	4 (4%)
I was too shy to report the injury (injuries)	3 (3%)
There was a lack of time to report the injury (injuries)	2 (2%)
I did not think about it at the time, and had patients waiting	1 (1%)
I was told no action was needed	1 (1%)
It was a complicated reporting procedure	1 (1%)
The patients were not affected	1 (1%)
**Total**	**113 (100%)**

### Post-traumatic stress disorder (PTSD)

In total, 5.38% (*n* = 5) of respondents who had sustained a sharps injury answered three or more of the four PTSD questions positively. This suggests that these students showed signs of PTSD linked specifically to the sharps injury. In total 41 respondents answered ‘yes’ to at least one PTSD question which suggests that 44.09% of healthcare students who sustained a sharps injury suffered some psychological effect of the injury.

## Discussion

This is one of the few studies which has specifically investigated sharps injuries within a wide selection of healthcare students. The overall incidence rate of sharps injuries amongst healthcare students in this study was 13% (*n* = 93). Studies investigating specifically healthcare students found a range of between 14.8% (Papadopoli et al., 2019) in Nepal and 42.8% (Bhattarai et al., 2014) in Italy. The rate identified in this study compares well with previous research.

Studies have also found that nursing, dentistry and medical students account for most sharps injuries within healthcare students (Bhattarai et al., 2014; McCarthy and Britton, 2000). In this study, the incidence rates of sharps injuries for nursing students were 17% (Child), 14% (Adult), 11% (Mental Health) and 21% (Midwifery). This compares favourably with the 35% prevalence rate in nursing students (Xu et al., 2022) and the 45.3% (Bouya et al., 2020) prevalence rates in healthcare workers reported within recent systematic reviews. The incidence rate for dentistry students was 9% and this compares favourably to the worldwide incidence range of between 13% (Smith et al., 2006) in the West Indies to 43.1% (*n* = 59) (Fernandes et al., 2017) in Brazil. The incidence rate for medical students was 12% and this compares favourably to incidence rates vary between 29.5% (*n* = 188) (Marusic et al., 2017) in Serbia to 39.3% (*n* = 184) (Ghasemzadeh et al., 2015) in Iran. Most injured healthcare students were in the first three academic years of their education, with the sixth year being the least common timeframe. This could link to the inexperience of the students with handling sharps (Malik et al., 2019).

This study showed that podiatry students and occupational therapy students sustained sharps injuries. Even though the number of participants was small, this may be the first identification of sharps injuries in this group of healthcare students worldwide. Hence, more research into these specific healthcare students is required. A total of 16% of paramedicine students in this study sustained a sharps injury. There is a lack of evidence relating to paramedicine students worldwide. The one other study conducted by Jung (2019) found an incidence of 28.8% in Korean paramedicine students, whilst a study of trained paramedics identified that 12% had sustained sharps injuries during their career (Garus-Pakowska et al., 2017). This highlights the potential risk of sharps injuries for paramedicine students within their training and the need for more research into this under-explored student and professional group.

The most common device causing a sharps injury in this study was glass ampoules (39.6%), although various types of needles amalgamated accounted for 42.7% of sharps injures. There are numerous devices which can cause a sharps injury to healthcare students. Equipment involved in a sharps injury is occasionally generic to different types of student such as various forms of needles (Bhattarai et al., 2014), but is sometimes particular to specific healthcare students, such as dentistry students and a scaler (Smith et al., 2006). This was evident in this study where many types of healthcare students had injuries with glass ampoules and needles which are generic to many professions, but also specific equipment linked to that programme, that is, a podiatry student sustaining a sharps injury with a nail nipper. An unusual finding was Adult Nursing student sustaining a sharps injury with a patient’s egg piercer which is a previously unreported device. This highlights the fact that some sharps injuries can occur in healthcare students with devices which are not by definition medical devices. Additionally, healthcare students should have access to safer sharps when in simulation and in practice when these types of safer devices are available.

In this study, the most common time for a sharps injury was between 0600 and 1159, followed by the afternoon. Previous studies with nursing students (Hambridge et al., 2021) and dentistry students (Fernandes et al., 2017) found similarly that most sharps injuries occurred in the morning and afternoon. This relates to the fact that healthcare students may be more likely to be in placement or simulating at this time compared to later parts of the day and night.

The most reported causes of a sharps injury in this study were the equipment, carelessness and inexperience. Many other studies have shown carelessness (Malik et al., 2019; Papadopoli et al., 2019; Wicker and Rabenau, 2010) and inexperience (Bhattarai et al., 2014; Fernandes et al., 2017; Ghasemzadeh et al., 2015) to be causes of sharps injuries within healthcare students. This could relate to healthcare students failing to give attention to avoidable risks involved with sharps handling and management due to their potential lack of experience, awareness and knowledge. Equipment is not specifically mentioned within the literature but may come under the umbrella of inexperience and completing the skills for the first time. Stress was a rare cause within this study, although this has been more prevalent in other studies (Bhattarai et al., 2014; Wicker and Rabenau, 2010) causing 16.7% and 50% of sharps injuries, respectively. Also, the ‘uncooperative’ or ‘restless’ patient was not mentioned within this study, although it has been reported in many others (Bhattarai et al., 2014; Ghasemzadeh et al., 2015).

In total, more than one third of students (36.6% *n* = 34) were not being observed when the sharps injury occurred. This fits with other studies where between 21.4% of nursing students (Hambridge et al., 2021) and 41.2% of dental students (Wicker and Rabenau, 2010) sustained a sharps injury when not being observed. Not being observed is a contributing factor as this offers the healthcare student to potentially practice unsafely due to issues such as carelessness, time pressures and inexperience.

The most common location for a sharps injury to occur in this study was in the treatment room, the patient’s bedside and in a clinic. Reported locations where healthcare students sustain sharps injuries varies widely in the literature (Fernandes et al., 2017; Marusic et al., 2017). Some occur within the numerous practice placement settings (Shen et al., 1999), but also within university environments (Myers et al., 2012). The most common specialities where sharps injuries occurred in this study were medical placements, obstetrics/gynaecology/maternity, and the education simulation environment. Similarly, many various specialities have been reported within the literature (Ghasemzadeh et al., 2015) of where healthcare students sustain sharps injuries. Smith et al. (2006) first reported that nursing students sustain sharps injuries within clinical skills simulation. It is essential that healthcare students who may be anxious and inexperienced are supervised effectively within the simulation setting. It is here that learning occurs regarding the use of safe sharps and the importance of complying with guidance, policies and laws regarding sharps safety.

Most injuries occurred in this study to the hand, although one injury occurred to the lip and one to the arm. This links with previous research showing that between 81.1% (Papadopoli et al., 2019) and 97% (Shen et al., 1999) of sharps injuries occur to the hand, relating to the most common part of the body in contact with the sharp.

More than half of sharps injuries were not reported, with paramedicine students and dentistry students having the highest reporting rates (75% *n* = 6) and occupational therapy students having the lowest reporting rate (0% *n* = 1). Worrying, evidence suggests that many sharps injuries are not reported by healthcare students, with the non-reporting range being between 28.75% (Malik et al., 2019) within dental students in Pakistan and 71.5% (Wicker and Rabenau, 2010) in dental students in Germany. The most reported reasons in this study for not reporting the sharps injury included it being a minor injury and being unused or clean equipment. Research into medical students has found similar reasons (Marusic et al., 2017). The non-reporting of sharps injuries by healthcare students may mean missed opportunities for physical and psychological care, missed prophylactic treatments, and the non-identification of poor or unsafe practice which would need to be addressed through education.

In total, 5.38% (*n* = 5) of respondents who had sustained a sharps injury answered three or more of the four Post-Traumatic Stress Disease (PTSD) questions positively, suggesting that the sharps injury may have contributed to PTSD. This is similar to the PTSD rate identified by Hambridge et al. (2021) in nursing students who had sustained sharps injuries. This finding highlights the need for trained healthcare professionals and academics to be aware of the potential need for the psychological follow-up care for healthcare students who sustain a sharps injury.

## Conclusion

This study identified that as well as nursing, medical and dentistry students, podiatry, midwifery, paramedicine and occupational therapy students sustain sharps injuries. The most common devices causing sharps injury was shown to be glass and needles, with the most frequent locations being the treatment room and the patient’s bedside. The equipment, carelessness and inexperience were stated as the contributing factors to sharps injuries. The hand was the most common site of the body affected, with the most common specialities being medicine and obstetrics/gynaecology/maternity. Some students showed signs and symptoms of PTSD following the sharps injury. Further research needs to be performed investigating not just the association between sharps injuries and PTSD, but sharps injuries within healthcare students other than nursing, medical and dentistry. There is scope for a review of the education strategies for healthcare students relating to the importance of using safe sharps, how to handle sharps, sharps management and the prevention of injuries.
